# Population-based pattern of medication use and prevalence of polypharmacy among patients with cardiovascular diseases: results of the Pars cohort study from Iran

**DOI:** 10.1186/s12872-022-02872-7

**Published:** 2022-10-06

**Authors:** Pooran Mohsenzadeh, Ali Ardekani, Hossein Poustchi, Zahra Mohammadi, Seyed Reza Abdipour Mehrian, Hamed Bazrafshan Drissi, Zahra Rahimian, Erfan Taherifard, Ali Nabavizadeh, Alireza kamalipour, Bita Mesgarpour, Fatemeh Malekzadeh, Hossein Molavi Vardanjani

**Affiliations:** 1 MPH Department, School of Medicine, Shiraz University of Medical Sciences, Shiraz, Iran; 2 Health Policy Research Center, Institute of Health, Shiraz University of Medical Sciences, Shiraz, Iran; 3 Liver and Pancreatobiliary Diseases Research Center, Digestive Diseases Research Institute, Tehran University of Medical Sciences, Tehran, Iran; 4 Department of Cardiology, School of Medicine, Shiraz University of Medical Sciences, Shiraz, Iran; 5 Student Research Committee, Shiraz University of Medical Sciences, Shiraz, Iran; 6 Department of Ophthalmology, Hamilton Glaucoma Center, Shiley Eye Institute, University of California, San Diego, CA USA; 7 Vice Chancellery for Research and Technology, National Institute for Medical Research and Development (NIMAD), Tehran, Iran; 8 Digestive Diseases Research Center, Digestive Diseases Research Institute, Shariati Hospital, Tehran University of Medical Sciences, Tehran, Iran; 9 MPH Department, School of Medicine, Research Center for Traditional Medicine and History of Medicine, Shiraz University of Medical Sciences, Shiraz, Iran

**Keywords:** Polypharmacy, Medication adherence, Cardiovascular diseases, Prevalence

## Abstract

**Background:**

Polypharmacy in patients with cardiovascular diseases (CVDs) has been linked to several adverse outcomes. This study aimed to investigate the pattern of medication use and prevalence of polypharmacy among CVDs patients in Iran.

**Method:**

We used the baseline data of the Pars cohort study (PCS). The participants were asked to bring their medication bags; then, the medications were classified using the Anatomical Therapeutic Chemical classification. Polypharmacy was defined as using five or more medications concurrently. Poisson regression modeling was applied. The adjusted prevalence ratios (PR) and its 95% confidence interval (CI) were estimated.

**Results:**

Totally, 9262 participants were enrolled in the PCS, of whom 961 had CVDs. The prevalence of polypharmacy in participants with and without CVDs was 38.9% and 7.1%, respectively. The highest prevalence of polypharmacy (51.5%) was among obese patients. Abnormal waist-hip ratio (PR: 2.79; 95% CI 1.57–4.94), high socioeconomic status (PR: 1.65; 95% CI 1.07–2.54), tobacco-smoking (PR: 1.35; 95% CI 1.00–1.81), patients with more than three co-morbidities (PR: 1.41; 95% CI 1.30–1.53), high physical activity (PR: 0.66; 95% CI 0.45–0.95), use of opiate ever (PR: 0.46; 95% CI 0.26–0.82), and healthy overweight subjects (PR: 0.22; 95% CI 0.12–0.39) were associated with polypharmacy. Cardiovascular drugs (76.1%), drugs acting on blood and blood-forming organs (50.4%), and alimentary tract and metabolism drugs (33.9%) were the most frequently used drugs. Agents acting on the renin-angiotensin system were the mostly used cardiovascular system drugs among men and those above 60 years old, while beta-blocking agents were mostly prevalent among cardiovascular system drugs in women with CVDs.

**Conclusion:**

Given the high prevalence of polypharmacy among CVDs patients, and subsequent complications, programs to educate both physicians and patients to prevent this issue is crucial.

## Introduction

The aging trend of the populations around the world has led to an increasing burden of non-communicable diseases (NCDs) [1], particularly cardiovascular diseases (CVDs). CVDs are the primary cause of mortality (one-third of deaths) and morbidity globally, affecting half of all individuals over their lifetime [2, 3]. Most of these patients suffer from other NCDs such as diabetes mellitus (DM), hypertension, dyslipidemia, and chronic kidney disease [4], which require concomitant administration of several drugs to control them. Although using multiple medications in these patients is inevitable for controlling the diseases, it may lead to improper use of the medications among a significant number of the patients [5, 6].

In various studies on different populations, polypharmacy prevalence among patients varied from over 10 to 90% [7–12]. Also, cardiovascular drugs are reported to be the most prevalent type of drugs in patients with polypharmacy [13, 14]. Simultaneous use of multiple medications in these patients may lead to administration difficulties, reduce adherence levels, and contribute to dosage errors [15, 16]. Polypharmacy is also associated with adverse events such as drug interactions, longer hospital stay, more frequent falls and fractures, excessive health expenditure, and increased mortality risk [7, 8, 14]. Hence, recognizing high-risk subgroups for polypharmacy in CVDs patients is vital to decrease the above-mentioned complications and lessen the burden of CVDs-related comorbidities.

Despite the significant burden of CVDs in developing countries, the knowledge regarding polypharmacy in CVDs patients is still limited [7, 17]. In the present investigation, we aimed to measure the prevalence and characteristics of polypharmacy among patients with CVDs in a cohort study in southern Iran.

## Methods

### Study setting

This cross-sectional study was conducted on the baseline data of the Pars Cohort Study (PCS). PCS is an ongoing population-based prospective study that started in 2012 to determine the prevalence of NCDs risk factors in a semi-urban area, Valashahr, in Fars province, South of Iran. Valashahr has over 40,000 inhabitants, of whom nearly 10,000 people are between 40 and 75 years old. Detailed information on the studied population was published elsewhere [18]. All Valashahr residents who were between 40 and 75 years old were contacted and invited to participate in PCS. Finally, 9264 individuals agreed to participate (92% participation rate). For the current analysis, two participants were excluded due to insufficient data. Trained medical personnel (physicians and nurses) collected the data, including in-person interviews, a brief physical examination, anthropometric indices measurements, and biomedical samples analysis using standardized and calibrated equipment.

### Data collection

The participants were questioned, “Has your physician told you that you have CVDs and need treatment for that?”. If the answer was “Yes”, they were categorized as having CVDs. They were asked to bring their bags of medications to the PCS center. An educated nurse listed the drugs (used at least over the past three months) and asked the patients which medications were taken at the time of the interview. Although various definitions have been proposed for polypharmacy [19], in the present investigation, polypharmacy was defined as the concurrent use of five or more different medications.

### Drug classification

Aside from complementary medicines, we utilized the first level of the Anatomical Therapeutic Chemical (ATC) classification system [20] to classify the participants’ medications. Also, to categorize cardiovascular drugs, we used ATC code C.

### Polypharmacy determinants

We defined the covariates as follows: age (40–49, 50–59, and 60 ≤), gender (female, male), education (literate, illiterate), marital status (married, not married), ethnicity (Fars/ non-Fars), tobacco smoking ever-use (smoker, non-smoker), central obesity based on waist to hip ratio [21] (with central obesity, without central obesity), fasting blood sugar (FBS > 110 mg/dL, FBS ≤ 110), low density lipoprotein-cholesterol (LDL˂100 mg/dL, LDL ≥ 100), high density lipoprotein-cholesterol (HDL < 45 mg/dL, HDL ≥ 45), total cholesterol (< 200 mg/dL, 200–239, and 240 ≤), triglyceride (< 150 mg/dL, 150–199, and 200 ≤), body mass index (BMI < 25; normal weight, 25–29.9; overweight, and 30 ≤ ; obese), and age at diagnosis of CVDs (< 40, 40–60, and ≥ 60).

Metabolic syndrome was defined according to the criteria suggested by Alberti et al. [22] for Asian individuals. Participants without metabolic syndrome (MetS) who were overweight were described as “healthy overweight”, and those who were overweight and had Mets simultaneously were considered as “unhealthy overweight”. The participants’ socioeconomic status (SES) was measured by applying their self-reported assets. Asset analysis was done by multiple correspondence analysis, and a latent factor was estimated. According to the quartiles of the estimated latent factor, we classified the participants into four groups (low, low-middle, middle-high, and high). Physical activity data were obtained through International Physical Activity Questionnaire (IPAQ) [23] and converted to Metabolic equivalent of task (MET) scores. Then, the participants were categorized into three groups including high (at least 3000 MET-minutes/week), moderate (at least 600 MET-minutes/week), and low (less than 600 MET-minutes/week).

We asked the participants about a list of diseases to determine comorbidities, “whether or not your doctor or health care provider has ever diagnosed each of these diseases for you?”. These conditions included hypertension, DM, jaundice, rheumatic heart disease, joint pain, back pain, anxiety, depression, insomnia, obstructive lung disease, stroke, renal failure, and cancer. Also, gastroesophageal reflux disease (GERD), irritable bowel syndrome (IBS), and functional constipation were defined according to previously described clinical criteria [24]. The participants were divided into three groups based on their comorbidities: only CVDs, two or three comorbidities, and more than three comorbidities. Furthermore, considering the date of the interview and the date of diagnosis of CVDs, we assessed the duration of CVDs for each patient (less than 2, 3 to 5 years, and 6 years and more).

### Statistical analysis

Frequency, mean, and standard deviation (SD) were calculated to describe the variables, where appropriate. Prevalence ratio (PR) and its 95% confidence interval (CI) were assessed using Poisson distribution. Considering the standard world population (WHO 2000–25), the age-standardized prevalence of polypharmacy was estimated. Chi-square and Mann–Whitney U tests were used for univariate analyses. To determine independent correlates of polypharmacy prevalence, Poisson regression modeling was applied; variables with a univariate p value of less than 0.3 were included in the multivariable modeling as potentially independent variables. In the final analysis, the model was proportionated using a backward elimination technique. p values less than 0.05 were considered statistically significant. STATA software (Release 11, College Station, TX:Stata Corp LLC) was used to analyze the data.

## Results

Of 9262 participants, 961 were CVDs patients, including 404 (42.0%) men and 557 (58%) women with the mean age of 58.5 ± 9.8. The prevalence of polypharmacy was 374/961 (38.9%, 95% CI 35.8%, 42.0%). The overall age- and gender-standardized prevalence of polypharmacy was 36.0% (95% CI 32.7%, 39.2%). The estimated age-standardized prevalence of polypharmacy was 29.6% (95% CI 25.1%, 34.1%) for males and 40.6% (95% CI 35.9%, 45.4%) for females.

**Fig. 1:**
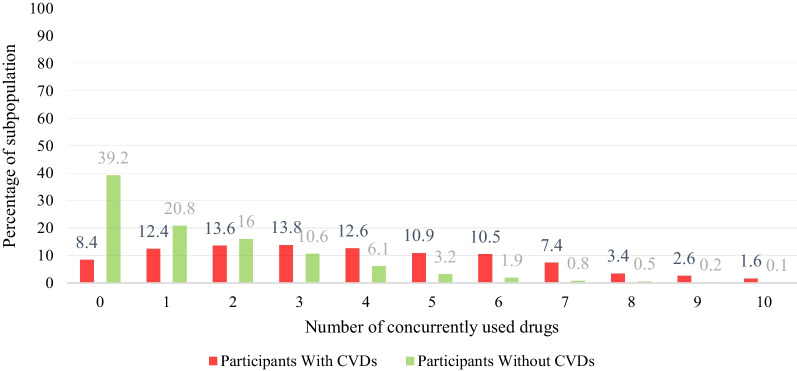
The number of concurrently used drugs categorized by having CVDs. *The numbers above each bar are the percentages

**Fig. 2:**
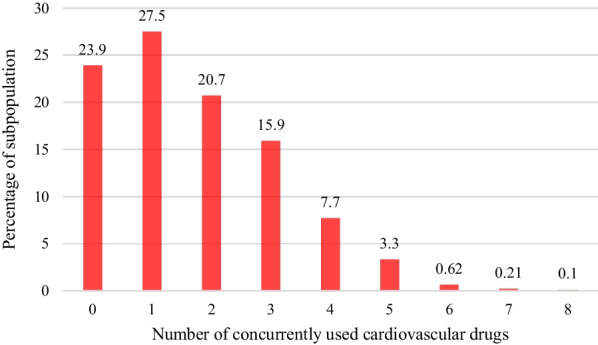
The number of concurrently used cardiovascular drugs used by patients with CVDs

Among the participants with CVDs, the highest estimated prevalence of polypharmacy (51.5%; 95% CI 44.9%, 58.1%) was among obese patients, and the lowest prevalence belonged to patients without central obesity (16.6%; 95% CI 10.6%, 25.1%). In all variables, the prevalence of polypharmacy was significantly higher in patients with CVDs compared to those without CVDs (Table 1). Also, Fig. 1 displays the percentage of concurrently used drugs among patients with and without CVDs, and Fig. 2 shows number of concurrently cardiovascular drugs used by patients with CVDs.

**Table 1:** Prevalence of polypharmacy and characteristics of patients with and without CVDs enrolled in the Pars cohort study

Characteristics*		Patients with CVDs		Patients without CVDs	
		*n* = 961 (100%)	Polypharmacy n (P%; 95% CI)	n = 8301 (100%)	Polypharmacy n (P%; 95% CI)
Overall		961 (100)	374 (38.9; 35.8–42.0)	8301 (100)	590 (7.1; 6.5–7.6)
*Gender*					
Male		404 (42.0)	120 (29.7; 25.4–34.3)	3871 (46.6)	84 (2.1; 1.7–2.6)
Female		557 (57.9)	254 (45.6; 41.4–49.7)	4430 (53.3)	506 (11.4; 10.5–12.3)
p			< 0.001		< 0.001
*Age (years)*					
40–49		187 (19.4)	55 (29.4; 23.3–36.3)	4022 (48.4)	257 (6.3; 5.6–7.1)
50–59		333 (34.6)	120 (36.0; 31–41.3)	2492 (30.0)	182 (7.3; 6.3–8.3)
60 +		441 (45.8)	199 (45.1; 40.5–49.8)	1787 (21.5)	151 (8.4; 7.2–9.8)
p			0.001		0.017
*Age at diagnosis*					
Less than 40		63 (6.5)	14 (22.2; 13.6–34.1)	–	
40–60		666 (69.3)	265 (39.7; 36.1–43.5)		
Above 60		232 (24.1)	95 (40.9; 34.7–47.4)		
p			0.018		
*Duration (years)*					
Less than 2		333 (34.8)	112 (33.6; 28.7–38.8)	–	
3–5		347 (36.3)	144 (41.4; 36.4–46.7)		
6 +		275(28.8)	112(40.7; 35–46.6)		
p			0.073		
*Education*					
Literate		330 (34.3)	115 (34.8; 29.8–40.1)	4394 (52.9)	285 (6.4; 5.7–7.2)
Illiterate		631 (65.6)	259 (41.0; 37.2–44.9)	3907 (47.0)	305 (7.8; 7.0–8.6)
p			0.061		0.019
*Marital status*					
Not married		145 (15.1)	65 (44.8; 36.9–52.9)	904 (10.8)	81 (8.9; 7.2–11.0)
Married		815 (84.9)	309 (37.9; 34.6–41.3)	7395 (89.1)	508 (6.8; 6.3–7.4)
p			0.116		0.021
*Ethnicity*					
Non-Fars		395 (41.1)	131 (33.1; 28.6–37.9)	3652 (43.9)	206 (5.6; 4.9–6.4)
Fars		566 (58.9)	243 (42.9; 38.9–47.0)	4649 (56.0)	384 (8.2; 7.5–9.0)
p			0.002		< 0.001
*Socio- economic status*					
Low		240 ( 25.0)	81 (33.7; 28.0–39.9)	2168 (26.2)	125 (5.7; 4.8–6.8)
Low–Middle		278 (28.9)	115 (41.3; 35.7–47.2)	2212 (26.74)	150 (6.7; 5.8–7.9)
Middle-High		204 (21.2)	87 (42.6; 36.0–49.5)	1839 (22.2)	137 (7.4; 6.3–8.7)
High		238 (24.7)	90 (37.8; 31.8–44.1)	2053 (24.8)	176 (8.5; 7.4–9.8)
p			0.194		0.004
*BMI (kg/m*^*2*^*)*					
Normal weight		364 (37.8)	113 (31.0; 26.4–35.9)	3736 (45.0)	177 (4.7; 4.1–5.4)
Over weight		378 (39.3)	148 (39.1; 34.3–44.1)	3064 (36.9)	238 (7.7; 6.8–8.7)
Obese		219 (22.8)	113 (51.5; 44.9–58.1)	1501 (18.0)	175 (11.6; 10.1–13.3)
p			< 0.001		< 0.001
*Central obesity*					
Yes		859 (89.3)	357 (41.5; 38.3–44.8)	6606 (79.5)	545 (8.2; 7.6–8.9)
No		102 (10.6)	17 (16.6;10.6–25.1)	1695 (20.4)	45 (2.6; 1.9–3.5)
p			< 0.001		< 0.001
*Physical activity*					
Low		434 (45.1)	201 (46.3; 41.6–51.0)	2626 (31.6)	270 (10.2; 9.1–11.5)
Moderate		305 (31.7)	112 (36.7; 31.4 -42.2)	2751 (33.1)	210 (7.9; 6.6–8.6)
High		222 (23.1)	61 (27.4; 22.0–33.7)	2924 (35.2)	110 (3.7; 3.1–4.5)
p			< 0.001		< 0.001
*Tobacco use*					
Ever		465 (48.5)	203 (43.6; 39.2–48.2)	3072 (37.0)	269 (8.7; 7.8–9.8)
Never		492 (51.4)	169 (34.3; 30.2–38.6)	5217 (62.9)	320 (6.1; 5.5–6.8)
p			0.003		< 0.001
*Triglyceride (mg/dL)*					
Less than150		524 (54.5)	186 (35.4; 31.5–39.6)	5097 (61.4)	317 (6.2; 5.5–6.9)
150 and above		437 (45.4)	188 (43.0;38.4–47.7)	3204 (38.6)	273 (8.5; 7.6–9.5)
p			0.017		< 0.001
*Cholesterol (mg/dL)*					
Less than 200		554 (57.6)	232 (41.8; 37.8–46.0)	4774 (57.5)	323 (6.7; 6.0–7.5)
200 and above		407 (42.3)	142 (34.8; 30.4–39.6)	3527 (42.4)	267 (7.5; 6.7–8.4)
p			0.028		0.159
Abnormal LDL					
No		460 (47.8)	215 (46.7; 42.2–51.3)	3556 (42.8)	262 (7.3; 6.5–8.2)
Yes		501 (52.1)	159 (31.7; 27.8–35.9)	4745 (57.1)	328 (6.9; 6.2–7.6)
p			< 0.001		0.031
*Abnormal HDL*					
No		808 (84.0)	314 (38.8; 35.5–42.2)	7123 (85.8)	526 (7.3; 6.7–8.0)
Yes		153 (15.9)	60 (39.2; 31.7–47.1)	1178 (14.2)	64 (5.4; 4.2–6.8)
p			0.934		0.016
*Elevated FBS*					
No		714 (74.3)	247 (34.5; 31.1–38.1)	6918 (83.3)	413 (5.9; 5.4–6.5)
Yes		247 (25.7)	127 (51.4; 45.1–57.6)	1383 (16.6)	177 (12.7; 11.1–14.6)
p			< 0.001		< 0.001

*BMI* body mass index; *LDL* Low-density lipoprotein; *HDL* high-density lipoprotein; *FBS* fasting blood sugar

^*^For all variables, the prevalence of polypharmacy was significantly different between participants with and without CVDs

Overall, more than half of the patients (59.4%) suffered from a minimum of three comorbidities (p < 0.001). According to Table 2, the top three comorbidities with a significantly higher prevalence of polypharmacy compared to patients without those comorbidities were DM (62.9%; 95% CI 55.5%, 69.7%), obstructive lung disease (55.8%; 95% CI 43.9%, 67.1%), and hypertension (53.6%; 95% CI: 47.8%, 58.3%).

**Table 2:** Comorbidities among CVDs patients in the PCS

Variables		Total, n = 961 (100%)	Polypharmacy n (P%*; 95% CI**)	p value
Comorbidity	One	71 (7.4)	11 (15.4; 8.7–25.8)	< 0.001
	Two or three	319 (33.2)	83 (26.0; 21.4–31.1)	
	More than 3	571 (59.4)	280 (49.0; 44.9–53.1)	
Hypertension	No	547 (56.9)	152 (27.7; 24.1–31.6)	< 0.001
	Yes	414 (43.1)	222 (53.6; 47.8–58.3)	
Diabetes miletus	No	783 (81.5)	262 (33.4; 30.2–36.8)	< 0.001
	Yes	178 (18.5)	112 (62.9; 55.5–69.7)	
Jaundice	No	926 (96.4)	358 (38.6; 35.5–41.8)	0.401
	Yes	35 (3.6)	16 (45.7; 30.2–62.1)	
Rheumatic heart disease	No	954 ( 99.3)	371 (38.8; 35.8–42.0)	0.830
	Yes	7 (0.7)	3 (42.8; 14.3–77.0)	
Joint pain	No	315 (32.8)	95 (30.1; 25.3–35.4)	< 0.001
	Yes	646 (67.2)	279 (43.1; 39.4–47.0)	
Back pain	No	398 (41.4)	129 (32.4; 27.9–37.1)	0.001
	Yes	563 (58.6)	254 (43.5; 39.4–47.6)	
Anxiety	No	531 (55.3)	163 (30.6; 26.9–34.7)	< 0.001
	Yes	430 (44.7)	211 (49.0; 44.3–53.7)	
Depression	No	654 (68.1)	214 (32.7; 29.2–36.4)	< 0.001
	Yes	307 (31.9)	160 (52.1; 46.5–57.6)	
Insomnia	No	698 (72.6)	257 (36.8; 33.3–40.4)	0.030
	Yes	263 (27.4)	117 (44.4; 38.5–50.5)	
Obstructive lung disease	No	893 (92.9)	336 (37.6; 34.5–40.8)	0.003
	Yes	68 (7.1)	38 (55.8; 43.9–67.1)	
Stroke	No	910 (94.7)	350 (38.4; 35.3–41.6)	0.220
	Yes	51 (5.3)	24 (47.0; 33.8–60.6)	
Renal failure	No	941 (97.9)	364 (38.6; 35.6–41.8)	0.304
	Yes	20 (2.1)	10 (50.0; 29.3–70.6)	
GERD	No	630 (65.6)	232 (36.8; 33.1–40.6)	0.066
	Yes	331 (34.4)	142 (42.9; 37.6–48.3)	
IBS	No	803 (84.5)	300(37.3; 34.0–40.7)	0.028
	Yes	147 (15.5)	147 (46.9; 39.0–55.0)	
Functional constipation	No	841 (87.5)	316 (37.5; 34.3–40.9)	0.024
	Yes	120 (12.5)	58 (48.3; 39.5–57.2)	
Cancer	No	947 (98.5)	371 (39.1; 36.1–42.3)	0.176
	Yes	14 (1.5)	3 (21.4; 7.0–49.4)	

*GERD* gastroesophageal reflux disease

*Prevalence was estimated in row

**Confidence interval

According to Table 3, high levels of physical activity (Adjusted PR: 0.66; 95% CI 0.56, 0.78), opiate ever used (Adjusted PR: 0.46; 95% CI 0, 26, 0.82), and being healthy overweight (Adjusted PR: 0.22; 95% CI 0.12, 0.39) were associated with the lower prevalence of polypharmacy.

**Table 3:** Correlates of the prevalence of polypharmacy in CVDs patients

Factors	Adjusted *PR(95% CI)	Crude PR (95%CI)
Having abnormal waist -hip ratio	2.79 (1.57–4.94)	3.54 (2.06–6.06)
SES (Ref: Low level)		
Low–Middle	1.56 (1.04–2.33)	1.38 (0.96–1.98)
Middle–High	1.89 (1.23–2.92)	1.45 (0.99–2.14)
High	1.65 (1.07–2.54)	1.19 (0.82–1.73)
Having more than three co-morbidities (Ref: less than three co-morbidities)	1.41 (1.30–1.53)	1.46 (1.35–1.58)
Being tobacco smoker ever (Ref: no use)	1.35 (1.00–1.81)	1.48 (1.14–1.92)
Having high levels of physical activity	0.66 (0.45–0.95)	0.66 (0.56–0.78)
Being opiate user ever (Ref: no use)	0.46 (0.26–0.82)	0.36 (0.22–0.61)
Being healthy overweight (Ref: unhealthy overweight)	0.22 (0.12–0.39)	0.24 (0.14–0.42)

*Adjusted for age and gender

Cardiovascular drugs (76.1%), drugs acting on blood and blood-forming organs (50.4%), and alimentary tract and metabolism drugs (33.9%) were the most frequently used drugs among the participants with CVDs. Cardiovascular drugs (83.0%) and drugs acting on blood and blood-forming organs (55.1%) had the highest prevalence of use among the elderly. These two drug classes also had equally high prevalence rates among females and males, about 75.0% for cardiovascular drugs and 50.0% for drugs acting on blood and blood-forming organs (Table 4). According to Table 5, agents acting on the renin-angiotensin system (Class C09) were the mostly used cardiovascular system drugs among men and those above 60 years old, while beta blocking agents (Class C07) were the most common drugs used among cardiovascular system drugs in women.

**Table 4:** The first ATC classification of drugs used by patients with cardiovascular disease

Drug class	Total n = 961 (100%)	Men (Total n = 404) n (%; 95% CI)	Women (Total n = 557) n (%; 95% CI)	60 years and older (Total n = 441) n (%; 95% CI)
C: Cardiovascular system	731 (76.1)	295 (73.0; 64.9–81.9)	436 (78.3; 71.1–86.0)	366 (83.0; 74.7–92.0)
B: Blood and blood-forming organs	484 (50.4)	204 (50.5; 43.8–57.9)	280 (50.3; 44.5–56.5)	243 (55.1; 48.4–62.5)
A: Alimentary tract and metabolism	326 (33.9)	107 (26.5; 21.7–32.0)	219 (39.3; 34.3–44.9)	169 (38.3; 32.8–44.6)
G: Genitourinary system	278 (28.9)	12 (3.0; 1.5–5.2)	266 (47.8; 42.2–53.9)	98 (22.2; 18.0–27.1)
N: Nervous system	223 (23.2)	74 (18.3; 14.4–23.0)	149 (26.8; 22.6–31.4)	105 (23.8; 14.5–28.8)
H: Systemic hormonal preparations*	166 (17.3)	52 (12.9; 9.6–16.9)	114 (20.5; 16.9–24.6)	82 (18.6; 14.8–23.1)
M: Musculoskeletal system	119 (12.4)	27 (6.7; 4.4–9.7)	92 (16.5; 13.3–20.3)	71 (16.1; 12.6–20.3)
R: Respiratory system	67 (7.0)	23 (5.7; 3.6–8.5)	44 (7.9; 5.7–10.6)	42 (9.5; 6.9–12.9)
J: Anti-infectives for systemic use	16 (1.7)	7 (1.7; 0.7–3.6)	9 (1.6; 0.7–3.1)	4 (0.9; 0.3–2.3)
S: Sensory organs	10 (1.0)	1 (0.3; 0.0–1.4)	9 (1.6; 0.7–3.1)	7 (1.6; 0.6–3.3)

*Excluding sex hormones and insulin

**Table 5:** The second ATC classification level of cardiovascular system drugs used by patients with CVDs

Drug class	Total n = 961 (100%)	Men (Total n = 404) n (%; 95% CI)	Women (Total n = 557) n (%; 95% CI)	60 years and older (Total n = 441) n (%; 95% CI)
C09	362 (37.6)	143 (35.4; 29.8–41.7)	219 (39.3; 34.2–44.8)	189 (42.8; 36.9–49.4)
C07	344 (35.8)	121 (29.9; 24.8–35.7)	223 (40.0; 34.9–45.6)	165 (37.4; 31.9–43.5)
C01	291 (30.2)	128 (31.6; 26.4–37.6)	163 (29.2; 24.9–34.1)	173 (39.2; 33.6–45.5)
C10	266 (27.6)	118 (29.2; 24.1–34.9)	148 (26.5; 22.4–31.2)	133 (30.1; 25.2–35.7)
C08	150 (15.6)	39 (9.6; 6.8–13.2)	111 (19.9; 16.3–24.0)	99 (22.4; 18.2–27.3)
C03	80 (8.3)	39 (9.6; 6.8–13.2)	41 (7.3; 5.2–9.9)	45 (10.2; 7.4–13.6)
C05	16 (1.6)	7 (1.7; 0.7–3.5)	9 (1.6; 0.7; 3.0)	6 (1.3; 0.5–2.9)
C02	7 (0.7)	4 (0.9; 0.2–2.5)	3 (0.5; 0.1–1.5)	5 (1.1; 0.3–2.6)

*ATC* anatomical therapeutic chemical; *C01* cardiac therapy; *C02* antihypertensives; *C03* diuretics; *C05* vasoprotectives; *C07* beta blocking agents; *C08* calcium channel blockers; *C09* agents acting on the renin-angiotensin system; *C10* lipid modifying agents

## Discussion

This study found that more than one-third of CVDs patients are likely to suffer from polypharmacy. The prevalence of polypharmacy was nearly 1.5-fold higher among females. Having a higher SES, being physically inactive, using tobacco, not using opiates, number of chronic comorbidities, being unhealthy overweight, and having an abnormal waist-hip ratio have been shown to be associated with increased prevalence of polypharmacy in patients with CVDs.

The prevalence of polypharmacy in our study was higher than that in a study from Ethiopia among CVDs patients [9]. On the other hand, some studies reported a higher prevalence of polypharmacy than our results. For instance, in Qatar and Oman 75.5% and 76.3% polypharmacy prevalence rates were observed, respectively [7, 25]. Also, previous reports from developed countries, Switzerland (11.8%), and United Kingdom (22.8%) indicated a lower prevalence of polypharmacy [26, 27]. One reason for the discrepancies could be the designs of the studies and the studied populations; the study in Oman was conducted at a tertiary care hospital, the study from Ethiopia evaluated outpatients, and our study was a population-based one. Furthermore, the prevalence of chronic diseases like DM and hypertension, as the risk factors of CVDs, in Arabic countries was much higher than that in our population, [28], resulting in higher polypharmacy rates.

It was shown that the female gender was associated with a higher prevalence of polypharmacy. Al-Dahshan et al. discovered the same pattern in CVDs patients [25]. However, some studies have reported no impact of gender on polypharmacy [7, 8]. Sechana et al. have reported an opposite result, indicating that polypharmacy was higher in the male gender [29]. As to the higher prevalence of polypharmacy in females, being more concerned about their health status than men may lead to more health-seeking behaviors, higher rates of adherence to medication therapy, and self-medication [30, 31]. Another explanation may be the higher life expectancy and longer time period of living with chronic diseases [32] which subsequently contribute to higher medication use. Thus, a higher prevalence of polypharmacy was seen in the females in our study.

Compared to low SES, our results demonstrated that higher SES levels lead to a higher rate of polypharmacy. Such findings have also been reported by others [14]. In contrast, some studies have found no difference based on SES [7, 9, 25]. In the city where our study was conducted, specialized medical services are not available, so individuals with higher SES may have better accessibility and affordability to utilize diagnostic and treatment services in neighboring cities, which leads to receiving more medications than the others.

We found that physical inactivity, existence of unhealthy overweight, abnormal waist-hip ratio, and tobacco smoking were associated with a higher prevalence of polypharmacy in CVDs patients. These conditions are contributing factors in the development of NCDs [33] and metabolic syndrome which leads to higher odds of having CVDs [34], confirming the notion that individuals with more comorbidities are at higher risk of suffering from polypharmacy. Also, one possible reason is that these conditions make it harder to control the underlying diseases, which leads to more medication use. Another finding of our study was that opiate use led to a lower prevalence of polypharmacy. It may stem from lower adherence to prescribed therapies in individuals with substance abuse, such as opiates [35]. Another possible justification could be the sedative and strong analgesic effect of opiates, which lead to delays in the diagnosis of diseases in these subgroups.

As addressed in previous studies, we also investigated the relationship between the number of comorbidities and polypharmacy [8, 36]. It is important to emphasize that the higher number of comorbidities correlates with an increase in the prevalence of polypharmacy. This can be explained by multiple medications prescription to control several diseases. Over-prescription and polypharmacy can contribute to an increased risk of adverse drug-drug and drug-disease interactions [37]. One possible solution could be the use of polypills, multiple drugs in one tablet [38], which should be further investigated in future studies.

According to our results, the most commonly used drugs in the CVDs patients were those acting on the cardiovascular system (Class C), followed by blood and blood-forming organs medications (Class B) and those acting on the alimentary tract and metabolism (Class A). Al-Hashar et al. [7] in Oman and Al-Amin et al. [8] in Bangladesh have shown that among CVDs patients, drugs related to the alimentary tract and metabolism were the most frequently used after cardiovascular medications. One possible explanation for this may be the use of different medicines in patients with CVDs which makes it inevitable to use antacid drugs to control the GI upset caused by simultaneous consumption of other medications [39].

Among cardiovascular system drugs, we showed that agents acting on the renin-angiotensin system and beta blocking agents (BB) were the mostly used drugs by CVDs patients. BBs were the topmost used drug in females with a subtle difference. A study from Ethiopia reported that diuretics and angiotensin-converting-enzyme inhibitors were the most frequently used drugs among CVDs patients [9]. Agents acting on the renin-angiotensin system have several benefits like reducing blood pressure and proteinuria, being drug of choice for hypertension in DM, and decreasing cardiovascular mortality and morbidity [40]. Also, BBs are used in a wide range of cardiovascular diseases [41]. Although they are not used as the first-line treatment in hypertension, their wide usage in myocardial infarction, congestive heart failure, cardiac arrhythmias, coronary artery disease, and other conditions may justify its high prevalence in our study.

Our study has several strengths. It is a population-based ongoing cohort conducted in Iran among patients with CVDs, assessing polypharmacy. Our sample size allowed for statistical analysis with sufficient statistical power. Additionally, to reduce recall bias, we asked the patients to bring their medications with them, and a trained nurse checked the list of drugs. However, the study is not free of limitations. First, the cross-sectional design of the study prevents us from establishing a cause and effect relationship between the variables. Second, the temporality between time-dependent variables like obesity and polypharmacy could not be addressed. Also, our study did not discuss the effects of CVDs relapse or untreated CVDs on polypharmacy. Moreover, we asked the patients whether they were diagnosed with CVDs; this might lead to recall and confirmation bias.

## Conclusion

The prevalence of polypharmacy was high in patients with CVDs. Higher SES, physical inactivity, tobacco use, existence of several comorbidities, being unhealthy overweight, and abnormal waist-hip ratio were important predictors of polypharmacy in patients with CVDs. Training at physician and patient levels is crucial to inhibit the increasing trend of polypharmacy and subsequent complications.

## Data Availability

Data is available upon request by the PCS central board and the corresponding author. (https://persiancohort.com/).

## Abbreviations

NCDs: Non-communicable diseases
CVDs: Cardiovascular diseases
DM: Diabetes mellitus
PCS: Pars cohort study
LDL: Low density lipoprotein-cholesterol
HDL: High density lipoprotein-cholesterol
BMI: Body mass index
Mets: Metabolic syndrome
SES: Socioeconomic status
IPAQ: International Physical Activity Questionnaire
MET: Metabolic equivalent of task
GERD: Gastroesophageal reflux disease
IBS: Irritable bowel syndrome
SD: Standard deviation
CI: Confidence interval
PR: Prevalence ratio
BB: Beta blocking agents
